# Association Between Outdoor Air Pollution and Risk of Malignant and Benign Brain Tumors: The Multiethnic Cohort Study

**DOI:** 10.1093/jncics/pkz107

**Published:** 2020-01-03

**Authors:** Anna H Wu, Jun Wu, Chiuchen Tseng, Juan Yang, Salma Shariff-Marco, Scott Fruin, Timothy Larson, Veronica W Setiawan, Shahir Masri, Jacqueline Porcel, Jennifer Jain, Thomas C Chen, Daniel O Stram, Loïc Le Marchand, Beate Ritz, Iona Cheng

**Affiliations:** 1 Department of Preventive Medicine, Keck School of Medicine, University of Southern California, 1441 Eastlake Ave, Rm 4443, Los Angeles, CA 90089, USA; 2 Program in Public Health, Susan and Henry Samueli College of Health Sciences, University of California, Anteater Instruction & Research Bldg (AIRB) # 2034, 653 East Peltason Drive, Irvine, CA 92697-3957, USA; 3 Department of Epidemiology and Biostatistics, University of California, 550 16th Street, Box 0560, San Francisco, CA 94158, USA; 4 Department of Civil & Environmental Engineering, University of Washington, 269 Wilcox Hall Box352700, School of Public Health, Seattle, WA 98195, USA; 5 Frontdoor Inc, 150 Peabody Place, Memphis, TN 38103, USA; 6 Department of Neurological Surgery, Keck School of Medicine, University of Southern California, GNH 3300, Mail code, Los Angeles, CA 90089-9314, USA; 7 Epidemiology Program, University of Hawaii Cancer Center, 701 Ilalo Street. Honolulu, HI 96813, USA; 8 Department of Epidemiology, School of Public Health, University of California, 650 Charles Young Dr. South, Los Angeles, CA 90095-1772, USA

## Abstract

**Background:**

There are increasing concerns about the potential impact of air pollution on chronic brain inflammation and microglia cell activation, but evidence of its carcinogenic effects is limited.

**Methods:**

We used kriging interpolation and land use regression models to estimate long-term air pollutant exposures of oxides of nitrogen (NO_x_, NO_2_), kriging interpolation for ozone (O_3_), carbon monoxide, and particulate matter (PM_2.5_, PM_10_), and nearest monitoring station measurements for benzene for 103 308 men and women from the Multiethnic Cohort, residing largely in Los Angeles County from recruitment (1993–1996) through 2013. We used Cox proportional hazards models to examine the associations between time-varying pollutants and risk of malignant brain cancer (94 men, 116 women) and meningioma (130 men, 425 women) with adjustment for sex, race and ethnicity, neighborhood socioeconomic status, smoking, occupation, and other covariates. Stratified analyses were conducted by sex and race and ethnicity.

**Results:**

Brain cancer risk in men increased in association with exposure to benzene (hazard ratio [HR] = 3.52, 95% confidence interval [CI] = 1.55 to 7.55) and PM_10_ (HR = 1.80, 95% CI = 1.00 to 3.23). Stronger associations with PM_10_ (HR = 3.02, 95% CI = 1.26 to 7.23), O_3_ (HR = 2.93, 95% CI = 1.09 to 7.88), and benzene (HR = 4.06, 95% CI = 1.17 to 18.2) were observed among Latino men. Air pollution was unrelated to risk of meningioma except that O_3_ exposure was associated with risk in men (HR = 1.77, 95% CI = 1.02 to 3.06). Brain cancer risk in women was unrelated to air pollution exposures.

**Conclusions:**

Confirmation of these sex differences in air pollution–brain cancer associations and the stronger findings in Latino men in additional diverse populations is warranted.

The etiology of malignant brain cancer remains largely unknown; only ionizing radiation and a history of allergies or atopic disease have been consistently associated with risk (1–3). In 2012, the International Agency for Research on Cancer classified air pollution and particulate matter (PM) as human carcinogens (4). Air pollutants may reach the brain via the systemic circulation for agents that can cross the blood brain barrier (5–7). However, epidemiological evidence of an association between exposure to outdoor air pollution and brain cancer is limited to results of mainly whites from one US cohort study of brain cancer mortality (8,9) and four primarily northern European [one case-control (10) and three cohort] studies on brain cancer incidence (11–13). These studies provided statistically nonsignificant associations for brain cancer with exposure to nitrogen oxide (NO_x_) (11) and PM_2.5absorbance_ (12) and no consistent associations for benign brain tumors (10,12,13). Volatile organic compounds such as benzene have been implicated in ecological studies of adult brain cancer (14–16) and in case-control studies of childhood brain cancer (17,18), but exposure to ambient benzene was not investigated.

Air pollution may adversely affect the central nervous system (CNS) by increasing the risk of stroke and decreasing cerebral blood flow and cognitive function (19–21), but research on the carcinogenic effects of air pollution on malignant and benign brain tumors has lagged behind. We conducted a large study to investigate the prospective effects of long-term exposure to NO_x_ and nitrogen dioxide (NO_2_), ozone (O_3_), carbon monoxide (CO), and benzene as well as PM_2.5_ and PM_10_ on risk of malignant brain cancer and meningioma within the California (CA) component of the Multiethnic Cohort (MEC) in which Latinos and African Americans represent approximately 75% of the participants. To our knowledge, this is the first prospective study on airborne air pollutants and risk of malignant and benign brain tumors with a substantial number of nonwhites and an assessment of sex-specific associations.

## Materials and Methods

### Study Participants

The MEC enrolled 96 810 men and 118 441 women aged 45–75 years from five racial and ethnic groups (African American, Japanese American, Latino, Native Hawaiian, and white) residing in Hawaii (HI) or CA (primarily Los Angeles County [LAC]) in 1993–1996 (22). At baseline, participants completed a 26-page questionnaire that assessed demographic, diet, anthropometrics, reproductive history, and lifestyle factors.

### Ascertainment of Malignant Brain Cancers and Meningioma

Participants were followed prospectively for incident invasive brain cancer through linkage with the CA and HI statewide cancer registries, a part of the Surveillance, Epidemiology, and End Results Program, and for vital status through linkages to the National Death Index and death certificate files. MEC participants older than 65 years were linked to Centers for Medicare Services claims (1999–2016) (23). To ascertain meningioma, we first used a subpopulation of MEC participants who were linked to Medicare data using established methods (23). We also identified meningioma case patients using cancer registry information on nonmalignant brain tumors (meninges spinal cord and other CNS tumors became a reportable disease on January 1, 2004) (24) (Supplementary Methods, available online).

Eligible CA participants completed a baseline questionnaire and provided valid addresses that were geocoded at the parcel or street segment level across the study period (n = 105 616). We excluded participants with a brain cancer or meningioma diagnosis before cohort entry and those with questionable address data (n = 2189) and other invalid entry or exit dates (n = 41), leaving 103 308 men and women for linkage with air pollution data. This cohort was followed from the date of entry (1993–1996) to the earliest date of diagnosis of brain cancer or meningioma, death, or December 31, 2013 (study end date), whichever came earlier (mean ± SD follow-up time = 16.4 ± 5.4 years). The study was approved by the institutional review boards at all participating institutions.

### Address History, Geocoding, and Air Pollution Exposure Assessment

The MEC actively maintains accurate and up-to-date addresses via periodic mailings of newsletters, follow-up questionnaires, and linkages to administrative data and registries. For the 103 308 CA MEC participants included in this study, 178 714 addresses were recorded during the follow-up period.

Air pollution exposure assessment methods in the MEC using kriging interpolation, land use regression (LUR), and the California Line Source Dispersion Model (CALINE4) have been published (25). Kriging interpolation estimated largely regional gaseous and PM pollutant exposures based on routine continuous air-monitoring data (26). The LUR models estimated regional and local NO_2_ and NO_x_ exposures based on data from spatially dense air-monitoring campaigns (27). The CALINE4 model estimated local concentrations of NO_x_ based on traffic emissions data within 1500 meters of a residential location and meteorological and roadway data (28). The US Environmental Protection Agency’s measured monthly benzene data from 1993 to 2016 were used, considering the availability of data for each month and year (ie, level of missing data) and the proximity of air monitors to the participants’ residential addresses. Valid data were defined as nonmissing data derived from monitors less than 20 km from residential addresses, and as such, benzene had low missing (3%) and invalid (7%) data. For sensitivity analyses, we evaluated benzene estimates derived from monitors less than 15 km, less than 10 km, and less than 7 km from residential addresses (Supplementary Methods, available online).

### Statistical Analysis

We employed time-dependent approaches to assess air pollutant exposures, capturing the variability over time and the differing duration of exposures across participants, to evaluate their associations on risk. For every participant’s address across the study period, we calculated a set of averages of cumulative exposures for a series of time intervals based on monthly (kriging and LUR) or yearly (CALINE4) exposures at cohort entry and each month and year during the follow-up until the censoring month and year (ie, diagnosis of brain cancer or meningioma, death, or study end). Averages of cumulative exposures were entered into Cox proportional hazard models as time-dependent variables via the counting process style of input. For the regression calculation, the average exposure starting from entry time until the time of the event was used for risk calculations, using age at cohort entry (5-year age categories) as a stratum variable and adjusted for covariates at baseline and yearly estimates of neighborhood socioeconomic status (nSES) at baseline and at time of event. Hazard ratios (HRs) and 95% confidence intervals (CIs) for a standard unit increase in an air pollutant were calculated; these common fixed units were chosen to be close to the respective interquartile ranges (Supplementary Methods, available online). To examine the independent effects of pollutants (Supplementary Table 1, available online), we conducted copollutant models by mutually adjusting for benzene and kriged O_3_ and PM_10_ overall and by sex.

Deviations from proportionality were checked using an analysis of Schoenfeld residuals; we found no violation of this assumption. Stratified analyses were conducted by sex, race and ethnicity, and distance to major roads because associations may differ by these factors (25,29,30). We tested for heterogeneity by including an interaction term in the model for each pollutant with these variables as applicable. Obesity, hypertension, and hormonal factors (women only) were associated with meningioma risk in the MEC (31), and thus all analyses were adjusted for these risk factors as well as for smoking and occupation because they represent potential sources of exposure to benzene and other pollutants (32,33). Of the 210 malignant brain cancers included in this analysis, 165 were gliomas (C71.0-C71.9) and 35 were cancers of the spinal cord or cranial nerves (C72.0-C72.9). We repeated analyses restricted to gliomas only.

All statistical tests were based on two-tailed probability and a significance level set α less than .05.

## Results

The mean ± SD age at study enrollment was 61.3 ± 9.3 years for men and 60.5 ± 8.5 years for women (Table 1). Latinos (47.6% men, 37.9% women) and African Americans (27.0% men, 35.9% women) represented about 75% of the CA MEC participants and the remaining were Japanese Americans (13.7% men, 10.6% women) or whites (11.6% men, 15.4% women). One-quarter of men (23.2%) and women (25.6%) lived in the lowest nSES (quintile 1). Men were more likely to have worked in industries as laborers or craftsmen (18.3%) than women (4.9%) and to have ever smoked (71.4% vs 46.2%, respectively).

**Table 1.** Distribution of study population characteristics in the MEC, at baseline, 1993–1996

Baseline characteristics	Male	Female
Mean age at entry (SD), y	61.3 (9.3) 42.5	60.5 (8.5) 57.3
	(N = 43 880) N(%)	(N = 59 218) N(%)
Age, y		
≤54	10 076 (23.0)	15 891 (26.8)
55–64	16 378 (37.3)	22 092 (37.3)
65–74	16 150 (36.8)	19 669 (33.2)
≥75	1276 (2.9)	1566 (2.6)
Race and ethnicity		
African American	11 832 (27.0)	21 239 (35.9)
Hawaiian	92 (0.2)	77 (0.1)
Japanese	6009 (13.7)	6301 (10.6)
Latino	20 870 (47.6)	22 466 (37.9)
White	5077 (11.6)	9135 (15.4)
Education*		
≤High school	21 633 (49.3)	31 124 (52.6)
Some college	12 390 (28.2)	16 819 (28.4)
College graduate	4957 (11.3)	5344 (9.0)
Graduate or professional	4224 (9.6)	4966 (8.4)
Baseline nSES*		
Quintile 1 (low)	10 192 (23.2)	15 133 (25.6)
Quintile 2	11 162 (25.4)	15 603 (26.3)
Quintile 3	8813 (20.1)	11 743 (19.8)
Quintile 4	7957 (18.1)	10 163 (17.2)
Quintile 5	5726 (13.0)	6555 (11.1)
Occupation†		
No industry, office or professional	15 838 (36.1)	28 346 (47.9)
No industry, labor or craftsman	6659 (15.2)	6839 (11.5)
No industry, office and or labor or craftsman	7707 (17.6)	19 342 (32.7)
Yes industry, office or professional	3319 (7.6)	1067 (1.8)
Yes industry, labor or craftsman	8025 (18.3)	2910 (4.9)
Yes industry, missing occupation	2332 (5.3)	714 (1.2)
History of high blood pressure		
No	25 816 (58.8)	33 848 (57.2)
Yes	18 064 (41.2)	25 370 (42.8)
Body mass index (kg/m^2^)*		
<25	13 696 (31.2)	20 960 (35.4)
25–<30	21 539 (49.1)	20 648 (34.9)
≥30	8188 (18.7)	16 536 (27.9)
Smoking history*		
Never smoker	12 560 (28.6)	31 873 (53.8)
Ex-smoker, <20 pack-years	15 424 (35.2)	13 448 (22.7)
Ex-smoker, 20+ pack-years	5149 (11.7)	2508 (4.2)
Ex-smokers, pack-years unknown	1444 (3.3)	1303 (2.2)
Current smoker, <20 pack-years	4758 (10.8)	5729 (9.7)
Current smoker, 20+ pack-years	3582 (8.2)	2584 (4.4)
Current smoker, pack-year unknown	235 (0.5)	190 (0.3)
Use of birth control pills		
No	NA	47 923 (80.9)
Yes		11 295 (19.1)
Age at first live birth, y*		
<20		21 290 (36.0)
21–30		25 477 (43.0)
>30		3387 (5.7)
Menopausal status*		
Premenopause	NA	5870 (9.9)
Natural menopause		28 721 (48.5)
Surgical menopause (oophorectomy)		8724 (14.7)
Other surgical (hysterectomy)		11 909 (20.1)
Period stopped, unknown reason		3228 (5.5)
Use of hormone replacement therapy		
Never E, with or without past or current P	NA	31 955 (54.0)
Past E, with or without past P		10 611 (17.9)
Current E use alone		6594 (11.1)
Current E use with P- past or current		6272 (10.6)
No. of residential moves over follow-up		
0	27 167 (61.9)	34 491 (58.2)
1–2	13 276 (30.3)	19 242 (32.5)
3–5	3239 (7.4)	5133 (8.7)
6+	198 (0.5)	352 (0.6)
Distance from residence to major road, m^‡^		
<500	14 054 (32.0)	18 374 (31.0)
≥500	29 826 (68.0)	40 844 (69.0)

* Numbers may not total to 100% due to missing. E = estrogen; MEC = Multiethnic Cohort; P = progestin.

† Yes industry means participant had worked in one or more of these industries (metal production or processing; mining or quarrying; cotton, wool, or textile; plastic production or processing; gasoline refining or distribution; chemical production or use; rubber or tire manufacturing; shipyard work; farming; furniture making or woodworking; automobile repair; pesticide production; paint production or use).

‡ Major roads classified according to US Census: A1 (primary roads, typically interstate highways, with limited access, division between the opposing directions of traffic, and defined exits), A2 (primary major, noninterstate highways and major roads without access restrictions), and A3 (smaller, secondary roads, usually with more than two lanes).

Based on a total of 210 malignant brain cancers, there were no statistically significant associations for kriged pollutants on risk, with the strongest increase in risk estimated for O_3_ exposure (HR = 1.54, 95% CI = 0.95 to 2.51) (Table 2). There was no evidence for heterogeneity in risks by sex, but all six kriged pollutants were positively associated with risk of malignant brain cancer in men (HRs ranged from 1.14 for NO_x_ to 2.45 for CO) but not in women. In men, a hazard ratio of 1.80 (95% CI = 1.00 to 3.23) for PM_10_ was observed. Modelled estimates of LUR NO_2_ and NO_x_ and CALINE4 NO_x_ did not suggest any increase in risk for malignant brain cancer (Supplementary Table 2, available online).

**Table 2.** Risk of malignant brain cancer and exposure to kriging determined gaseous and particulate matter air pollutants and benzene

	All			Men			Women			
	Case/cohort	HR (95% CI)*	*P* ^†^	Case/cohort	HR (95% CI)^‡^	*P* ^†^	Case/cohort	HR (95% CI)^§^	*P* ^†^	Phet by sex^‖^
Kriging^¶^										
NO_x_	210/99 140	.87 (.51 to 1.51)	.63	94/42 356	1.14 (0.49 to 2.67)	.76	116/56 784	0.70 (0.34 to 1.45)	.34	0.68
NO_2_	210/100 598	1.09 (.56 to 2.11)	.80	94/42 896	2.21 (0.77 to 6.38)	.14	116/57 702	0.64 (0.27 to 1.48)	.29	0.35
O_3_	210/100 622	1.54 (.95 to 2.51)	.08	94/42 905	1.77 (0.87 to 3.60)	.11	116/57 717	1.40 (0.72 to 2.74)	.33	0.53
CO	210/10 0621	1.33 (.45 to 3.88)	.61	94/42 906	2.45 (0.45 to 13.3)	.30	116/57 715	0.83 (0.21 to 3.34)	.79	0.67
PM_10_	210/100 622	1.24 (.84 to 1.82)	.28	94/42 907	1.80 (1.00 to 3.23)	.05	116/57 715	0.91 (0.54 to 1.51)	.70	0.36
PM_2.5_	210/100 556	1.27 (.49 to 3.30)	.63	94/42 873	2.19 (0.49 to 9.78)	.31	116/57 683	0.80 (0.23 to 2.78)	.73	0.87
Distance from benzene monitors#										
<20 km	199/96 637	1.65 (.98 to 2.78)	.06	90/41 207	3.52 (1.55 to 7.55)	<.01	109/55 430	0.88 (0.45 to 1.75)	.72	0.11
<15 km	180/91 109	1.74 (1.06 to 2.86)	.03	80/38 632	2.73 (1.27 to 5.86)	.01	100/52 477	1.19 (0.82 to 2.27)	.60	0.46
<10 km	112/53 999	1.57 (1.03 to 2.40)	.04	44/23 362	2.15 (0.89 to 5.18)	.09	68/30 637	1.47 (0.68 to 3.16)	.32	0.54
<7 km	57/25 969	1.28 (.57 to 2.86)	.55	25/11 503	1.62 (0.42 to 6.27)	.48	32/14 466	1.05 (0.38 to 2.80)	.92	0.81
Benzene <20 km from monitors										
Distance from roads										
<500 m	64/30 410	2.44 (1.01 to 5.92)	.05	31/13 220	6.78 (1.77 to 26.0)	<.01	33/17 190	0.89 (0.28 to 2.85)	.85	0.09
≥500 m	135/66 227	1.38 (.72 to 2.67)	.33	59/27 987	2.48 (0.90 to 6.86)	.08	76/38 240	0.89 (0.38 to 2.10)	.80	0.46
Phet by distance**			0.29			0.17			0.94	

* All brain cancers included 165 malignant glioma (C71.0-C71.9) and 35 cancers of the spinal cord or cranial nerves (C72.0-C72.9). Stratified by age at entry, adjusted for race and ethnicity, sex, education, baseline body mass index, hypertension, nSES at baseline, current nSES, smoking, occupation, and for women also included oral contraceptive use, type of menopause, hormone replacement therapy, and age at first live birth. Categories of the covariate variables are shown in Table 1. CI = confidence interval; CO = carbon monoxide; HR = hazard ratio; NO_2_ = nitrogen dioxide; NO_x_ = nitrogen oxide; nSES = neighborhood socioeconomic status; O_3_ = ozone; Phet = *P* value for heterogeneity; PM_2.5_ = particular matter with a diameter of 2.5 µm or less ; PM_10_ = cparticulate matter with a diameter of 10 µm or less.

^†^ Two-sided *P* value for a given air pollutant was obtained from the Cox proportional hazards model with adjustment as specified for all participants combined and in men and women separately.

^‡^ Stratified by age at entry, adjusted for race and ethnicity, education, baseline body mass index, hypertension, nSES at baseline, current nSES, smoking, and occupation.

^§^ Stratified by age at entry, adjusted for race and ethnicity, education, baseline body mass index, hypertension, nSES at baseline, current nSES, smoking, occupation, oral contraceptive use, type of menopause, hormone replacement therapy, and age at first live birth.

^‖^ Two-sided *P* value for heterogeneity by sex for a given air pollutant was obtained by including an interaction term for each pollutant with sex.

^¶^ Hazard ratios calculated per 50 ppb NO, per 20 ppb NO_2_, per 10 ppb of O_3_, per 1000 ppb CO, and per 10 µg/m^2^ of PM_2.5_ and PM_10_.

^#^ Hazard ratios calculated per 1 ppb benzene, within less than 20 km, less than 15 km, less than 10 km, and less than 7 km distance from monitoring stations.

** Two-sided *P* value for heterogeneity by distance from road was obtained by including an interaction term for benzene with distance.

In men and women combined, hazard ratios of malignant brain cancer increased with exposure to benzene (HR = 1.65, 95% CI = 0.98 to 2.78) and this was again due to an elevated risk in men (HR = 3.52, 95% CI = 1.55 to 7.55) but not in women (Table 2). The benzene to risk association in men was strongest in never smokers (HR = 6.02, 95% CI = 1.37 to 26.41), intermediate in former smokers (HR = 4.15, 95%CI = 1.21 to 14.22), and weakest in current smokers (HR = 1.09, 95% CI = 0.17 to 6.95). Distances of residences from major roads were unrelated to risk (per 200 m HR = 1.00, 95% CI = 0.95 to 1.05) in men and 0.99 (95% CI = 0.95 to 1.04) in women (data not shown). However, the benzene to risk association was stronger among men and women who lived less than 500 m from major roads (HR = 2.44, 95% CI = 1.01 to 5.92) than those who lived more than 500 m away (HR = 1.38, 95% CI = 0.72 to 2.67) (*P* for heterogeneity  = 0.29) (Table 2). We conducted sensitivity analyses using a stricter distance requirement that considered participants who lived within 15 km, 10 km, and 7 km of benzene monitoring stations; the respective hazard ratios were 1.74 (95% CI = 1.06 to 2.86), 1.57 (95% CI = 1.03 to 2.40), and 1.28 (95% CI = 0.57 to 2.86) in men and women combined (Table 2).

Brain cancers in Latino men and women accounted for about one-half of the malignant brain cancers. In race or ethnic specific analyses, Latino men displayed a statistically significant increased risk in relation to PM_10_ (HR = 3.02, 95% CI = 1.26 to 7.23), O_3_ (HR = 2.93, 95% CI = 1.09 to 7.88), and benzene (HR = 4.06, 95% CI = 1.17 to 18.2), but this was not observed in Latino women (Table 3). Benzene exposure was associated with a statistically nonsignificant increased risk in non-Latino men (Supplementary Table 3, available online).

**Table 3.** Risk of malignant brain cancer and exposure to kriging determined gaseous and particulate matter air pollutants and benzene in Latinos

	All Latino			Latino men			Latino women			
	Case/cohort	HR (95% CI)*	*P* ^†^	Case/cohort	HR (95% CI) ^‡^	*P* ^†^	Case/cohort	HR (95% CI) ^§^	*P* ^†^	Phet by sex^‖^
Kriging^¶^										
NO_x_	102/42 585	1.34 (0.52 to 3.48)	.55	46/20 474	0.82 (0.22 to 3.13)	.78	56/22 111	2.09 (0.52 to 8.46)	.30	0.89
NO_2_	102/42 609	2.09 (0.64 to 6.88)	.22	46/20 484	3.40 (0.56 to 20.7)	.18	56/22 125	1.31 (0.27 to 6.40)	.74	0.11
O_3_	102/42 623	1.42 (0.64 to 3.15)	.39	46/20 491	2.93 (1.09 to 7.88)	.03	56/22 132	0.57 (0.15 to 2.20)	.41	0.06
CO	102/42 624	1.08 (0.89 to 1.32)	.45	46/20 492	1.04 (0.79 to 1.38)	.30	56/22 132	1.12 (0.83 to 1.49)	.46	0.90
PM_10_	102/42 623	1.60 (0.85 to 3.00)	.15	46/20 492	3.02 (1.26 to 7.23)	.01	56/22 131	0.87 (0.35 to 2.18)	.77	0.03
PM_2.5_	102/42 586	1.03 (0.21 to 5.13)	.97	46/20 473	2.19 (0.49 to 9.78)	.31	56/22 113	0.77 (0.09 to 6.65)	.81	0.75
Benzene <20 km of monitors#	97/41 284	2.73 (1.10 to 6.74)	.03	43/19 830	4.06 (1.17 to 18.2)	.03	54/21 452	1.55 (0.43 to 5.55)	.51	0.03

* Stratified by age at entry, adjusted for sex, education, baseline body mass index, hypertension, nSES at baseline, current nSES, smoking, occupation, and for women also included oral contraceptive use, type of menopause, hormone replacement therapy, and age at first live birth. Categories of the covariate variables are shown in Table 1. CI = confidence interval; CO = carbon monoxide; HR = hazard ratio; NO_2_ = nitrogen dioxide; NO_x_ = nitrogen oxide; nSES = neighborhood socioeconomic status; O_3_ = ozone; Phet = *P* value for heterogeneity; PM_2.5_ = particular matter with a diameter of 2.5 µm or less; PM_10_ = particulate matter with a diameter of 10 µm or less.

^†^ Two-sided *P* value for a given air pollutant was obtained from the Cox proportional hazards model with adjustment as specified for all participants combined, and in men and women separately.

^‡^ Stratified by age at entry, adjusted for education, baseline body mass index, hypertension, nSES at baseline, current nSES, smoking, and occupation.

^§^ Stratified by age at entry, adjusted for education, baseline body mass index, hypertension, nSES at baseline, current nSES, smoking, occupation, oral contraceptive use, type of menopause, hormone replacement therapy, and age at first live birth.

^‖^ Two-sided *P* value for heterogeneity by sex for a given air pollutant was obtained by including an interaction term for each pollutant with sex.

^¶^ Hazard ratios calculated per 50 ppb NO, per 20 ppb NO_2_, per 10 ppb of O_3_, per 1000 ppb CO, and per 10 µg/m^2^ of PM_2.5_ and PM_10_.

^#^ Hazard ratios calculated per 1 ppb benzene, within less than 20 km distance from monitoring stations.

The associations between risk of gliomas only and exposure to kriging pollutants and benzene were similar to all malignant brain cancers, but almost all hazard ratios were slightly stronger for glioma only. The largest increase in hazard ratios was in relation to NO_2_ exposure in men (HR = 3.37, 95% CI = 0.99 to 11.5) (Supplementary Table 4, available online).

The air pollutants measured at the closest monitor (benzene) and modeled by kriging, LUR, and CALINE4 were moderately to strongly correlated (Supplementary Table 1, available online). In copollutant models conducted in men, the effects of both benzene and O_3_ remained statistically significant, whereas only benzene but not PM_10_ remained statistically significant. In copollutant models of kriged PM_10_ and O_3_, the PM_10_ association remained statistically significant (HR = 1.79, 95% CI = 1.03 to 3.11) (Table 4).

**Table 4.** Risk of malignant brain cancer in association with exposure to benzene and kriging pollutants in copollutant models

	All			Men			Women		
	Case/cohort	HR (95% CI)*	*P* ^†^	Case/cohort	HR (95% CI)‡	*P* ^†^	Case/cohort	HR (95% CI)^§^	*P* ^†^
Copollutant^‖^									
Benzene	199/96 633	1.84 (1.08 to 3.15)	.03	90/41 204	4.28 (1.92 to 9.55)	<.01	109/55 429	0.86 (0.42 to 1.76)	.69
O_3_		1.58 (0.82 to 3.06)	.17		3.07 (1.22 to 7.73)	.02		0.86 (0.33 to 2.20)	.52
Benzene	199/96 634	1.48 (0.84 to 2.63)	.18	90/41 205	3.10 (1.33 to 7.2)	<.01	109/55 429	0.80 (0.37 to 1.72)	.56
PM_10_		1.31 (0.83 to 2.06)	.25		1.53 (0.76 to 3.08)	.23		1.22 (0.67 to 2.24)	.52
Benzene	199/96 635	1.66 (0.87 to 3.16)	.13	90/41 205	4.30 (1.66 to 1.12)	<.01	109/55 430	0.67 (0.28 to 1.65)	.39
CO		1.01 (0.87 to 3.16)	.94		0.93 (0.75 to 1.15)	.49		1.10 (0.90 to 1.35)	.34
PM_10_	210/99 140	1.25 (0.87 to 1.84)	.34	94/42 901	1.79 (1.03 to 3.11)	.04	116/57 112	0.93 (0.56 to 1.54)	.78
O_3_		1.58 (0.97 to 2.58)	.07		1.84 (0.90 to 3.76)	.10		1.39 (0.71 to 2.72)	.34
									

* Stratified by age at entry, adjusted for race and ethnicity, sex, education, baseline body mass index, hypertension, nSES at baseline, current nSES, smoking, occupation, and for women also included oral contraceptive use, type of menopause, hormone replacement therapy, and age at first live birth. Categories of the covariate variables are shown in Table 1. CI = confidence interval; CO = carbon monoxide; HR = hazard ratio; nSES = neighborhood socioeconomic status; O_3_ = ozone; PM_10_ = particulate matter with a diameter of 10 µm or less.

^†^ Two-sided *P* value for given air pollutant was obtained from the Cox proportional hazards model with copollutants and with adjustment as specified for all participants combined, and in men and women separately.

^‡^ Stratified by age at entry, adjusted for race and ethnicity, education, baseline body mass index, hypertension, nSES at baseline, current nSES, smoking, and occupation.

^§^ Stratified by age at entry, adjusted for race and ethnicity, education, baseline body mass index, hypertension, nSES at baseline, current nSES, smoking, occupation, oral contraceptive use, type of menopause, hormone replacement therapy, and age at first live birth.

^‖^ Hazard ratios calculated per 1 ppb benzene, per 10 µg/m^2^ of PM_10,_ per 10 ppb of O_3_, and per 1000 ppb CO in copollutant models as specified.

Benzene and air pollutants determined by kriging (Supplementary Table 5, available online), LUR, and CALINE4 (Supplementary Table 2, available online) were unrelated to meningioma risk. There were no sex differences in these associations except that O_3_ was associated with meningioma risk in men (HR = 1.77, 95% CI = 1.02 to 3.06) but not in women (*P* value for heterogeneity by sex = 0.03). Benzene exposure based on less than 20 km to monitors was not associated with risk in men (HR = 1.20, 95% CI = 0.58 to 2.48), but this association became stronger when we restricted to residence less than 10 km of the monitoring stations (HR = 1.45, 95% CI = 1.02 to 2.08).

## Discussion

We observed increased risks of malignant brain cancer in relation to long-term exposure to benzene, O_3_, and possibly PM_10_ in men but not in women. There was little evidence for a link between these pollutants and meningioma risk. Our sex difference findings are novel, compatible with the higher population incidence and worse survival of brain cancer in men (34,35) and in line with sex difference findings in experimental studies (36–39).

We observed no association between malignant brain cancer risk and exposure to NO_x_ and NO_2,_ in agreement with previous studies (8–13). However, our findings of an increased risk of malignant brain cancer in men in relation to PM_10_ and O_3_ exposures differ from reports of no association with O_3_ (8,9), PM_10_, or PM_2.5_ (8,9,12) in men and women combined. Our stronger findings may be due to investigating sex-specific associations and the finding that associations were observed only in men. If future studies confirm this sex-specific association, then previous studies of women only (13) or men and women combined [women comprised 43–74% of brain cancers (10–12)] may have missed the sex-specific effect. In the Cancer Prevention Study II report, the only study that presented sex-specific results, PM_10_ was unrelated to mortality in both men and women, but consistent with our results there was a statistically nonsignificant increased risk with O_3_ in men but not in women (8).

Although the carcinogenicity of benzene to humans is well established (32,33), brain cancer risk has not been consistently associated with occupational exposures to benzene (40,41) or cigarette smoking, a major source of benzene exposure in humans (1). Published analytic studies on ambient benzene and malignant brain cancer in adults are lacking. Nevertheless, ambient benzene exposures have been linked to specific childhood brain tumors in a CA study that relied on measured concentrations from monitoring stations within 8 km of a residence (17) and a Texas study using the US Environmental Protection Agency’s modeled concentrations at Census tract level (18). We examined risk associations with ambient benzene exposure using four cutpoints of distances to monitors (<20 km, <15 km, <10 km, <7 km), which included, respectively, 93%, 88%, 52%, and 25% of study participants and found consistent risk associations at these distances. These benzene findings are noteworthy because the assessment of ambient benzene is challenging because of the lack of extensive measurement data and the rapid drop off in benzene from the emission sources (42). Ambient benzene originates from vehicle emissions, specifically incomplete fuel combustion and gasoline evaporation (43,44). Although benzene emissions have decreased by more than 70% in the past 2 decades in LAC, current benzene emissions are almost equally distributed between gasoline and nongasoline sources (45). Because of the small number of benzene monitors in LAC, our estimates are spatially coarse, capturing regional-scale variations of benzene from traffic-related sources but not microscale variations. It is intriguing that we found strong risk associations with benzene, which is related to traffic fuels but no associations with LUR and CALINE4 models of NO_x_ and NO_2_ that are expected to capture the finer spatial impacts of traffic emissions. This is consistent with our findings of higher correlations between benzene and kriged CO (*r* = 0.879) followed by kriged NO_2_ (*r* = 0.824) than with the LUR and CALINE4 modeled NO_x_ and NO_2_ (Supplementary Table 1, available online), indicating that our benzene exposure likely captured the regional rather than local impact of traffic emissions. A stronger association with benzene for residences less than 500 m from roadways (Table 2) may be due to coexposure to air toxics from local traffic emissions that follow similar exposure patterns as urban background benzene levels.

Our null results for meningioma agree with previous studies (10,12,13). The increased risk between O_3_ exposure and meningioma risk in men (HR = 1.77) may be a chance finding, but this result is compatible with our O_3_–malignant brain cancer finding in men.

A novel finding is the increased brain cancer risk in relation to exposure to O_3_, PM_10_, and benzene among men but not in women. Results in women were similar for those who had ever used hormone therapy and never users (data not shown). There are intriguing sex differences in benzene-induced toxicities in animal models that may be related to hormonal differences (46,47). Studies in mice exposed to diesel exhaust have shown microglia cell activation, increased neuroinflammation, and lipid peroxidation in all brain regions that were much worse in male than female mice (36,37). Brains of female mice may be less susceptible than those of male mice because of higher expression levels of paraoxonase 2 (*PON2*), an intracellular antioxidant and antineuroinflammatory enzyme that protects cells in the CNS; estrogens are important modulators of *PON2* (48). Fitting with our results, astrocytes of male compared with female mice have exhibited a greater susceptibility for tumor suppressor inactivation and an increased induction of a brain cancer stem cell phenotype (30,49).

Our study is the first to our knowledge to include a large number of nonwhites in an air pollution–brain cancer study, showing a more prominent risk in Latino men. Although a higher proportion of Latino men (35.7%) and women (35.6%) lived within 500 m of major roads compared with non-Latino men (28.7%) and women (28.1%), this factor alone is unlikely to explain the stronger association in Latino men that was absent in Latino women. Moreover, distance from major roads was not associated with risk in Latino men or women (all HRs were close to 1.0). Interactions with occupational or genetic risk factors and coexposures with other carcinogens in Latino men may be additional factors. Interestingly, a polymorphism in the *PON1* gene promoter region (108 C > T) was implicated in a study of glioma conducted in Mexico (50); this *PON1* allele is more frequent in Hispanics compared with whites (51).

Air pollution may have a causative role for malignant brain cancer via oxidative stress and neuroinflammation pathways. O_3_ inhalation may be associated with elevated circulating proinflammatory mediators that can access the CNS by crossing the blood-brain barrier (52), inducing inflammation within and outside the airways primarily via local generation of lipid ozonation products and resulting in extrapulmonary inflammatory responses. Prolonged exposures to PM and diesel exhaust have been associated with molecular changes in rodent brains that are consistent with the activation of brain tumor pathways (53). Specifically, exposure of rats to PM_2.5–10_ from LAC for 1–3 months was found to trigger the expression of inflammatory stress- and cancer-related biomarkers, including upregulation of *IL16*, *IL13-Rα1*, and *EGR2* genes in the brain (54). These genes have been implicated in astrocytoma and glioblastoma multiforme (55,56).

Strengths of this study include investigating both malignant and benign brain tumors in men and women of multiple races and ethnicities, large numbers of Latinos and African Americans, extensive information on risk factors and potential confounders, and the availability of long-term residential address history. We identified meningioma using two established national resources (Medicare and Surveillance, Epidemiology, and End Results Program registries).

However, as in other studies, we lacked information on exposures before cohort entry and during work and commuting. Given the long latency of brain cancer, historical air pollution exposures are likely etiologically relevant although the precise exposure window is unknown. Sensitivity analyses by length of follow-up and moving status yielded similar results (data not shown). Although we used established approaches (eg, kriging, LUR, CALINE4) to assess long-term air pollution exposures, the different approaches have varying levels of uncertainty pointing to imprecision of our estimates and the potential for nondifferential misclassification of individual exposures. Ambient benzene measurement is challenging. To our knowledge, no cancer studies, including ours, have long-term high spatiotemporal resolution estimates for benzene exposures. Although it is desirable to account for collinearity of pollutants beyond using simple mutual adjustment approaches, it is difficult to implement multipollutant approaches (eg, hierarchical clustering, hierarchical Bayesian) (57–59) to examine time-varying collinearity of copollutants and to estimate their effects on risk in a time-dependent manner. We did not have information on black carbon, which was implicated in the ESCAPE study based on PM_2.5_ absorbance (12). Finally, our modest sample size precluded investigation of tumor location and histological subtypes of malignant brain cancers. Our stronger findings restricted to gliomas only (Supplementary Table 4, available online) suggest air pollution exposures may be particularly important for the more aggressive brain cancers and that understanding etiological heterogeneity of brain tumor subtypes is needed.

In conclusion, this prospective cohort study found that men, especially Latino men, living in areas with high ambient benzene, O_3_, and PM_10_ levels experienced a higher risk of malignant brain cancer. Confirmation of these results is needed in additional well-designed studies including minority men and women and using long-term and improved spatiotemporal exposure assessment. This will help to better understand the role that ambient air pollution plays in malignant brain cancer development.

## Funding

This work was supported by the Health Effects of Air Pollution Foundation, the National Cancer Institute (U01 CA164973), USC Norris Comprehensive Cancer Center Core Support (P30 CA014089), Environmental Exposures, Host Factors, and Human Disease (P30 ES007048021), California Air Resource Board (contract 04–323), National Institute of Environmental Health Sciences (NIEHS R21 ES016379), Health Effect Institute (HEI 4787-RFA09-4110–3), and National Cancer Institute (5-R21-CA094723-03).

## Notes

The funders had no role in the design of the study; the collection, analysis, and interpretation of the data; the writing of the manuscript; and the decision to submit the manuscript for publication.

The authors are grateful to the study participants and research team of the MEC. The authors confirm they have no personal or financial conflicts of interest.

## Supplementary Material

pkz107_Supplementary_Data
