# Supplementary material for: Association Between Outdoor Air Pollution and Risk of Malignant and Benign Brain Tumors: The Multiethnic Cohort Study
Source: JNCI Cancer Spectr. 2020 Jan 3;4(2):pkz107. doi: 10.1093/jncics/pkz107 (PMC7083235; doi:10.1093/jncics/pkz107)
Supplement: pkz107_Supplementary_Data [file pkz107_supplementary_data.pdf]

Supplementary Methods

Ascertainment of Malignant Brain Cancers and Meningioma

Multiethnic Cohort (MEC) participants were followed prospectively for diagnosis of incident invasive brain cancer [malignant brain cancer, (C70.0, C71.0-C71.9), spinal cord and other CNS tumors (C72.0-C72.9) and histologies (9120-9161, 9380-9480, 9540-9560, 9370, 9494, 9505)] through routine linkage with the California Cancer Registry and the Hawaii Tumor Registry, which are a part of the National Cancer Institute's Surveillance, Epidemiology, and End Results Program (SEER), and for vital status through linkages to the National Death Index and death certificate files. MEC participants older than 65 years were linked to Centers for Medicare Services claims (1999-2016)[1]; 89% of these participants have been linked. The California participants were also linked to the California Office of Statewide Health Planning and Development (OSHPD) for hospital discharge data (1993-2014). To ascertain meningioma cases, we first utilized a subpopulation of MEC participants who have been linked to Medicare data using well-established methods [1]. Because participants who are enrolled in managed care plans were unlikely to have complete claim histories, the study population was restricted to California MEC fee-for-service participants. Second, we identified meningioma cases using cancer registry information on non-malignant brain tumors (meninges (C70.0-C70.9) and spinal cord and other CNS tumors (C72.0-C72.9)) that became a reportable disease on January 1, 2004 [2] according to the SEER mandated Benign Brain Tumor Cancer Registries Amendment Act. The International Classification of Diseases (ICD) 9th Revision codes 192.1, 192.3, 225.2, 225.4, and 237.6, and 10th Revision codes C70.0, C70.1, D32.0, and D32.1 were used from the Medicare hospitalization claim (MedPAR) or from OSHPD files to identify meningioma cases. Additional cases were identified from Medicare outpatient and carrier files if there were two or more claims with the ICD codes as above and the claims were greater than 30 days apart. In addition, we included tumors of the meninges (C70.0-C70.9) and meningioma histology codes

(9530-9539) [2]. We selected the earliest claim date from either Medicare or OSHPD as the discovery date. For meningioma diagnosis identified by both SEER and Medicare or OSHPD, we compared the date of diagnosis/identification (month/year) and used the earlier date of diagnosis/identification. Of the meningioma cases included in this analysis, 32.7% were identified by both SEER and Medicare/OSHPD, 56.0% from Medicare/OSHPD only, and 11.3% from SEER only.

Address History, Geocoding, and Air Pollution Exposure Assessment

For the 103,308 male and female MEC participants included in this study, there were 178,714 addresses recorded during the follow-up period. Residential addresses were geocoded to latitude and longitude coordinates of parcels, or street segments whenever parcels could not be identified. Geocoded addresses from 1993 through 2013 were linked to 1990 (1993-1996 addresses), 2000 (1997-2005 addresses), and 2010 (2006-2013 addresses) US Census block groups. Each MEC participant was assigned a composite measure of neighborhood socioeconomic status (nSES) [3, 4] based on the Census block group of his/her residential history across the study period that was categorized into quintiles based on the nSES distribution of Los Angeles County block groups.

Details of our air pollution exposure assessment methods for criteria air pollutants, using kriging interpolation, land use regression (LUR), and California line source dispersion model (CALINE4) have been presented recently [5]. In brief, kriging interpolation was used to estimate largely regional air pollution exposures for gaseous and PM pollutants based on routine NO_2, NO_x, O_3, CO, PM_{10}, and $PM_{2.5}$ [6]. Measured concentrations of NO_2, NO_x, PM_{10}, CO, and O_3 in 1993-2013 and $PM_{2.5}$ in 2000-2013 were obtained from US EPA routine air monitoring data. $PM_{2.5}$ concentrations in 1993-1999 were estimated from our published spatiotemporal model that used PM_{10}, meteorological and spatial variables [7]. The LUR models were used to estimate regional and local NO_2 and NO_x exposures based on air monitoring data from spatially

dense air monitoring campaigns and incorporated spatial predictors on land use and traffic characteristics; monthly scaling factors for temporal adjustment were applied based on routinely collected long-term air monitoring data nearest to the participant's home [8]. The CALINE4 model was used to estimate local concentrations of NO_x based on traffic emissions data within 1500 meters of a residential location and meteorological and roadway data [9]. The correlation coefficients between the air pollutants measured at the closest monitor (benzene) and modeled by kriging, LUR, and CALINE4 were similar in men and women and by race/ethnicity; we presented results for all subjects combined **(see Supplementary Table 1).**

Statistical Analysis

Hazard ratios (HRs) and corresponding 95% confidence intervals (CI) were calculated for each pollutant based on fixed units (50 ppb for NO_x, 20 ppb for NO_2,10 ppb for O_3, 1000 ppb for CO, 10 µg/m^3 for $PM_{2.5}$, 10 µg/m^3 for PM_{10} and 1 ppb for benzene). These fixed units were chosen to be close to the respective interquartile ranges (IQRs) of pollutants determined by LUR and kriging interpolation. For the pollutants determined by LUR, the IQRs were 41.7 ppb for NO_x and 18.2 ppb for NO_2. For the pollutants determined by kriging, the IQRs were 51.4 ppb for NO_x, 16.4 ppb for NO_2, 9.3 for O_3, 742.6 ppb for CO, 3.8 µg/m^3 for $PM_{2.5}$, and 9.0 µg/m^3 for PM_{10} and 1.2 ppb for benzene.

References

1. Setiawan VW, Virnig BA, Porcel J*, et al.* Linking data from the Multiethnic Cohort Study to Medicare data: linkage results and application to chronic disease research. Am J Epidemiol 2015;181(11):917-9.
2. Dolecek TA, Dressler EV, Thakkar JP*, et al.* Epidemiology of meningiomas post-Public Law 107-206: The Benign Brain Tumor Cancer Registries Amendment Act. Cancer 2015;121(14):2400-10.
3. Yost K, Perkins C, Cohen R*, et al.* Socioeconomic status and breast cancer incidence in California for different race/ethnic groups. Cancer Causes Control 2001;12(8):703-11.
4. Yang J, Schupp CW, Harrati A, Clarke Ca, Keegan THM, Gomez SL. Developing an area-based socioeconomic measure from American Community Survey data. Cancer Prevention Institute of California, Fremont, Calfornia. In; 2014.
5. Cheng I, Tseng C, Wu J*, et al.* Association between ambient air pollution and breast cancer risk: The Multiethnic Cohort Study. Int J Cancer 2019.
6. Laurent O, Hu J, Li L*, et al.* A Statewide Nested Case-Control Study of Preterm Birth and

Air Pollution by Source and Composition: California, 2001-2008. Environ Health Perspect 2016;124(9):1479-86.
7. Li L, Wu A, Cheng I*, et al.* Spatiotemporal estimation of historical PM2.5 concentrations using PM10, meteorological variables, and spatial effects. Atmos Environment 2017;166:182-191.
8. Su JG, Jerrett M, Beckerman B*, et al.* Predicting traffic-related air pollution in Los Angeles using a distance decay regression selection strategy. Environ Res 2009;109(6):657-70.
9. Wu J, Wilhelm M, Chung J*, et al.* Comparing exposure assessment methods for traffic-related air pollution in an adverse pregnancy outcome study. Environ Res 2011;111(5):685-92.

Supplementary Table 1. Pairwise correlation between air pollutant exposures by kriging, CALINE4, and LUR models, and benzene from baseline through study end date, MEC participants residing in California

**Pearson correlation coefficients**	Kriging NO_x	Kriging NO_2	Kriging O_3	Kriging CO	Kriging PM_{10}	Kriging $PM_{2.5}$	Caline4 NO_x	LUR NOx	LUR NO_2	Benzene
Kriging NO_x	1.000	0.902	-0.735	0.855	0.562	0.682	0.326	0.655	0.598	0.756
Kriging NO_2	_*	1.000	-0.556	0.852	0.783	0.732	0.284	0.601	0.706	0.824
Kriging O_3	_	_	1.000	-0.289	-0.117	-0.437	-0.259	-0.557	-0.504	-0.408
Kriging CO	_	_	_	1.000	0.601	0.447	0.286	0.523	0.541	0.879
Kriging PM_{10}	_	_	_	_	1.000	0.741	0.128	0.410	0.600	0.586
Kriging $PM_{2.5}$	_	_	_	_	_	1.000	0.215	0.449	0.479	0.413
Caline4 NO_x	_	_	_	_	_	_	1.000	0.597	0.517	0.247
LUR NOx	_	_	_	_	_	_	_	1.000	0.877	0.410
LUR NO_2	_	_	_	_	_	_	_	_	1.000	0.399
Benzene	_	_	_	_	_	_	_	_	_	1.000

* Results as shown

Supplementary Table 2. Risk of malignant brain cancer and meningioma and exposure to gaseous air pollutants determined by land use regression (LUR) and California Line Source Dispersion model (CALINE4)

	All			Men			Women			
Brain cancer	Case/ cohort	HR (95% CI) *	P value†	Case/ cohort	HR (95% CI) ‡	P value†	Case/ cohort	HR (95% CI) §	P value†	Phet by sex||
LUR¶										
NO_x	198/92025	0.82 (0.52-1.27)	0.37	87/39373	0.94 (0.49-1.82)	0.86	111/52652	0.71 (0.39-1.30)	0.27	0.86
NO_2	199/93354	0.86 (0.51-1.43)	0.55	87/39963	1.18 (0.56-2.49)	0.66	111/53391	0.62 (0.31-1.25)	0.18	0.62
CALINE4¶										
NO_x	191/97947	0.89 (0.29-2.73)	0.84	88/41590	1.05 (0.20-5.46)	0.95	103/56357	0.79 (0.17-3.65)	0.77	0.84
Meningioma										
LUR¶										
NO_x	503/92025	0.95 (0.70-1.27)	0.72	115/39373	0.77 (0.42-1.42)	0.40	388/52652	1.02 (0.73-1.43)	0.91	0.27
NO_2	513/93354	1.06 (0.75-1.50)	0.73	118/39963	1.00 (0.50-1.97)	0.99	392/53391	1.09 (0.73-1.62)	0.68	0.70
CALINE4 ¶										
NO_x	525/97957	1.52 (0.78-2.98)	0.22	119/41590	1.23 (0.30-5.11)	0.78	406/56357	1.63 (0.76-3.48)	0.21	0.51

* Stratified by age at entry, adjusted for race/ethnicity, sex, education, baseline body mass index, hypertension, neighborhood SES (nSES) at baseline, current nSES, smoking, occupation, and for women also included oral contraceptive use, type of menopause, hormone replacement therapy, and age at first livebirth. Categories of the covariate variables are shown in Table 1.

† Two-sided P value for a given air pollutant was obtained from the Cox proportional hazards model with adjustment as specified for all subjects combined, and in men and women separately.

‡ Stratified by age at entry, adjusted for race/ethnicity, education, baseline body mass index, hypertension, nSES at baseline, current nSES, smoking, and occupation

§ Stratified by age at entry, adjusted for race/ethnicity, education, baseline body mass index, hypertension, nSES at baseline, current nSES, smoking, occupation, oral contraceptive use, type of menopause, hormone replacement therapy, and age at first livebirth

|| Two-sided P value for heterogeneity by sex for a given air pollutant was obtained by including an interaction term for each pollutant with sex

¶ HRs calculated per 50 ppb NO_x and per 20 ppb NO_2

Supplementary Table 3. Risk of malignant brain cancer and exposure to kriging determined gaseous and particulate matter air pollutants and benzene in non-Latinos

	Non-Latino - All			Non-Latino Men			Non-Latino Women			
	Case/ cohort	HR (95% CI) *	P value†	Case/ cohort	HR (95% CI) ‡	P value†	Case/ cohort	HR (95% CI) §	P value†	Phet by sex||
Kriging¶										
NO_x	108/56555	0.70 (0.35-1.39)	0.31	48/21882	1.08 (0.33-3.56)	0.90	60/34673	0.54 (0.23-1.26)	0.16	0.61
NO_2	108/57989	0.84 (0.37-1.91)	0.68	48/22412	1.39 (0.35-5.53)	0.64	60/35577	0.60 (0.22-1.69)	0.34	0.91
O_3	108/57999	1.66 (0.88-3.12)	0.12	48/22414	1.33 (0.42-4.18)	0.63	60/35585	1.90 (0.89-4.05)	0.10	0.48
CO	108/57997	1.15 (0.30-4.39)	0.84	48/22414	2.04 (0.19-21.61)	0.55	60/35583	0.82 (0.16-4.20)	0.81	0.62
PM_{10}	108/57999	1.14 (0.70-1.85)	0.60	48/22415	1.37 (0.62-3.02)	0.44	60/35584	1.01 (0.55-1.85)	0.98	0.89
$PM_{2.5}$	108/57970	1.50 (0.44-5.14)	0.52	48/22400	2.85 (0.36-22.3)	0.32	60/35570	0.95 (0.20-4.47)	0.95	0.43
Benzene <20 km of monitors#	102/55353	1.28 (0.66-2.46)	0.46	47 /21375	2.38 (0.85-6.71)	0.10	55/33978	0.85 (0.37-1.93)	0.69	0.84

* Stratified by age at entry, adjusted for sex, race/ethnicity, education, baseline body mass index, hypertension, neighborhood SES (nSES) at baseline, current nSES, smoking, occupation, and for women also included oral contraceptive use, type of menopause, hormone replacement therapy, and age at first livebirth. Categories of the covariate variables are shown in Table 1.

† Two-sided P value for a given air pollutant was obtained from the Cox proportional hazards model with adjustment as specified for all subjects combined, and in men and women separately.

‡ Stratified by age at entry, adjusted for education, baseline body mass index, hypertension, nSES at baseline, current nSES, smoking, and occupation

§ Stratified by age at entry, adjusted for education, baseline body mass index, hypertension, nSES at baseline, current nSES, smoking, occupation, oral contraceptive use, type of menopause, hormone replacement therapy, and age at first livebirth

|| Two-sided P value for heterogeneity by sex for a given air pollutant was obtained by including an interaction term for each pollutant with sex

¶ HRs calculated per 50 ppb NO, per 20 ppb NO_2, per 10 ppb of O_3, per 1000 ppb CO, and per 10 µg/m^2 of $PM_{2.5}$ and PM_{10}

HRs calculated per 1 ppb benzene, within <20 km distance from monitoring stations

Supplementary Table 4. Risk of malignant glioma (C710-C719) and exposure to kriging determined gaseous and particulate matter air pollutants and benzene

	All			Men			Women			
	Case/ cohort	HR (95% CI) *	P value†	Case/ cohort	HR (95% CI) ‡	P value†	Case/ cohort	HR (95% CI) §	P value†	Phet by sex||
Kriging||										
NOx	165/99095	1.15 (0.61-2.15)	0.67	75/42337	1.31 (0.52-3.33)	0.57	90/56758	1.02 (0.43-2.41)	0.96	0.79
NO2	165/100553	1.65 (0.77-3.58)	0.20	75/42877	3.37 (0.99-11.5)	0.05	90/57676	0.95 (0.35-2.55)	0.91	0.28
O_3	165/100577	1.42 (0.82-2.44)	0.21	75/42886	1.74 (0.81-3.72)	0.16	90/57691	1.17 (0.53-2.58)	0.70	0.37
CO	165/100576	2.06 (0.60-7.16)	0.25	75/42887	3.02 (0.47-19.3)	0.24	90/57689	1.47 (0.27-7.92)	0.66	0.63
PM_{10}	165/100577	1.45 (0.94-2.23)	0.09	75/42888	1.97 (1.04-3.71)	0.04	90/57689	1.09 (0.61-1.94)	0.78	0.35
$PM_{2.5}$	165/100511	1.88 (0.63-5.66)	0.26	75/42854	2.61 (0.50-13.6)	0.26	90/57657	1.37 (0.32-5.98)	0.67	0.98
	Benzene <20 km of monitors¶									
All	159/96597	1.97 (1.09-3.58)	0.03	73/41190	3.87 (1.60-9.40)	<0.01	86/55407	1.08 (0.49-2.37)	0.85	0.11
<500m of roads	50/30396	2.63 (1.00-6.87)	0.05	24/13213	7.83 (1.62-37.9)	0.01	26/17183	1.07 (0.31-3.67)	0.92	0.10
≥500m of roads	109/66201	1.73 (0.81-3.68)	0.16	49/27977	2.85 (0.92-8.83)	0.07	60/38224	1.16 (0.42-3.23)	0.78	0.42
	Benzene <10 km of monitors#.									
All	89/53976	1.92 (0.98-3.75)	0.06	39/23357	2.57 (0.92-7.18)	0.07	50/30619	1.60 (0.64-4.02)	0.31	0.90
<500m of roads	25/17140	3.26 (0.77-13.8)	0.11	12/7514	4.18 (0.48-36.4)	0.20	13/9626	2.19 (0.25-19.6)	0.48	0.30
≥500m of roads	64/36836	1.52 (0.72-3.23)	0.27	27/15843	2.28 (0.65-7.95)	0.20	37/20993	1.43 (0.51-4.04)	0.50	0.41

* Of the 165 malignant gliomas, the top subsites were temporal lobe (C71.2, n=40), frontal lobe (C71.1, n=35), parietal lobe (C71.3, n=22) and overlapping sites (C71.8, n=38). Stratified by age at entry, adjusted for race/ethnicity, sex, education, baseline body mass index, hypertension, neighborhood SES (nSES) at baseline, current nSES, smoking, occupation, and for women also included oral contraceptive use, type of menopause, hormone replacement therapy, and age at first livebirth. Categories of the covariate variables are shown in Table 1.

† Two-sided P value for a given air pollutant was obtained from the Cox proportional hazards model with adjustment as specified for all subjects combined, and in men and women separately.

‡ Stratified by age at entry, adjusted for race/ethnicity, education, baseline body mass index, hypertension, nSES at baseline, current nSES, smoking, and occupation

§ Stratified by age at entry, adjusted for race/ethnicity, education, baseline body mass index, hypertension, nSES at baseline, current nSES, smoking, occupation, oral contraceptive use, type of menopause, hormone replacement therapy, and age at first livebirth

|| HRs calculated per 50 ppb NO, per 20 ppb NO_2, per 10 ppb of O_3, per 1000 ppb CO, and per 10 µg/m^2 of $PM_{2.5}$ and $PM_{10,}$

¶ HRs calculated per 1 ppb benzene, within <20 km distance from monitoring stations.

#. HRs calculated per 1 ppb benzene, within <10 km distance from monitoring stations.

Supplementary Table 5. Risk of meningioma and exposure to kriging determined gaseous and particulate matter air pollutants, and benzene

	All			Men			Women			
	Case/ cohort	HR (95% CI) *	P value†	Case/ cohort	HR (95% CI) ‡	P value†	Case/ cohort	HR (95% CI) §	P value†	Phet by sex||
Kriging¶										
NO_x	543/99140	0.88 (0.63-1.22)	0.44	128/42356	0.73 (0.39-1.39)	0.34	415/56784	0.93 (0.63-1.37)	0.70	0.25
NO_2	555/100598	1.02 (0.69-1.50)	0.94	130/42896	1.02 (0.46-2.25)	0.96	425/57702	0.99 (0.63-1.56)	0.97	0.25
O_3	555/100622	1.14 (0.84-1.55)	0.41	130/42905	1.77 (1.02-3.06)	0.04	425/57717	0.96 (0.66-1.39)	0.81	**0.03**
CO	555/100621	0.88 (0.47-1.66)	0.70	130/42906	0.79 (0.22-2.81)	0.71	425/57715	0.89 (0.43-1.83)	0.74	0.95
PM_{10}	555/100622	1.09 (0.88-1.35)	0.45	130/42907	1.27 (0.81-2.01)	0.30	425/57715	1.02 (0.80-1.31)	0.85	0.90
$PM_{2.5}$	555/100556	0.89 (0.55-1.45)	0.70	130/42874	0.87 (0.31-2.42)	0.79	425/57683	0.87 (0.50-1.52)	0.63	0.63
Benzene (distance from monitors) #										
<20 km	530/96637	0.91 (0.64-1.30)	0.61	121/41207	1.20 (0.58-2.48)	0.62	409/55430	0.83 (0.55-1.24)	0.35	0.90
<15 km	496/91109	1.14 (0.82-1.58)	0.43	109/38632	1.29 (0.64-2.60)	0.47	387/52477	1.09 (0.76-1.58)	0.66	0.42
<10 km	278/53999	1.18 (0.80-1.78)	0.41	59/23362	1.45 (1.02-2.08)	0.04	219/30637	1.01 (0.65-1.58)	0.96	0.49
<7 km	143/25969	1.15 (0.55-1.89)	0.95	30/11503	1.24 (0.32-4.79)	0.76	113/14466	0.97 (0.48-1.94)	0.92	0.41

* Stratified by age at entry, adjusted for race/ethnicity, sex, education, baseline body mass index, hypertension, neighborhood SES (nSES) at baseline, current nSES, smoking, occupation, and for women also included oral contraceptive use, type of menopause, hormone replacement therapy, and age at first livebirth. Categories of the covariate variables are shown in Table 1.

† Two-sided P value for a given air pollutant was obtained from the Cox proportional hazards model with adjustment as specified for all subjects combined, and in men and women separately.

‡ Stratified by age at entry, adjusted for race/ethnicity, education, baseline body mass index, hypertension, nSES at baseline, current nSES, smoking, and occupation

§ Stratified by age at entry, adjusted for race/ethnicity, education, baseline body mass index, hypertension, nSES at baseline, current nSES, smoking, occupation, oral contraceptive use, type of menopause, hormone replacement therapy, and age at first livebirth

|| Two-sided P value for heterogeneity by sex for a given air pollutant was obtained by including an interaction term for each pollutant with sex

¶ HRs were conducted per 50 ppb NO, per 20 ppb NO_2, per 10 ppb of O_3, per 1000 ppb CO, and per 10 µg/m^2 of $PM_{2.5}$ and PM_{10}

#. HRs were conducted per 1 ppb benzene, within <20 km, <15 km, <10 km, and <7 km from benzene monitoring stations
